# Frizzled receptor 6 marks rare, highly tumourigenic stem-like cells in mouse and human neuroblastomas

**DOI:** 10.18632/oncotarget.410

**Published:** 2011-12-31

**Authors:** Sandra Cantilena, Fabio Pastorino, Annalisa Pezzolo, Olesya Chayka, Vito Pistoia, Mirco Ponzoni, Arturo Sala

**Affiliations:** ^1^ Molecular Haematology and Cancer Biology Unit, UCL Institute of Child Health, 30 Guilford st London WC1N 1EH London UK; ^2^ Brunel Institute of Cancer Genetics and Pharmacogenomics, Brunel University, Uxbridge, Middlesex UB8 3PH, UK; ^3^ Laboratory of Oncology, G. Gaslini Institute, Largo G. Gaslini 5, 16147 Genova, Italy

**Keywords:** HIF, hypoxia, metastasis, neuroblastoma, stem cell

## Abstract

Wnt signalling is an important component of vertebrate development, required for specification of the neural crest. Ten Wnt receptors [Frizzled receptor 1-10 (Fzd1-10)] have been identified so far, some of which are expressed in the developing nervous system and the neural crest. Here we show that expression of one such receptors, Fzd6, predicts poor survival in neuroblastoma patients and marks rare, HIF1/2 α-positive cells in tumour hypoxic areas. Fzd6 positive neuroblastoma cells form neurospheres with high efficiency, are resistant to doxorubicin killing and express high levels of mesenchymal markers such as Twist1 and Notch1. Expression of Fzd6 is required for the expression of genes of the non-canonical Wnt pathway and the spheres forming activity. When transplanted into immunodeficient mice, neuroblastoma cells expressing the Fzd6 marker grow more aggressively than their Fzd6 negative counterparts. We conclude that Fzd6 is a new surface marker of aggressive neuroblastoma cells with stem cell-like features.

## INTRODUCTION

Neuroblastoma, the most common extracranial solid tumor in infancy, is an embryonic cancer originating from the neural crest [1]. The tumor develops in the sympathetic nervous system, typically the adrenal medulla or paraspinal ganglia, and is characterised by a wide spectrum of clinical behaviour. Localised neuroblastoma is curable, while metastatic neuroblastoma has a poor prognosis and high rate of relapse after chemotherapeutic treatments [1].

The neural crest is a multipotent cell population localized at the edge between the neural plate and non-neural ectoderm (epidermis). Neural crest cells migrate throughout the embryo and differentiate into several cell types, including neurons, chondroblasts and melanocytes. Molecular signals controlling neural crest specification are not fully understood but Wnt proteins, bone morphogenetic proteins (BMPs) and Fibroblast Growth Factors (FGFs) have all been associated with neural crest induction [2].

Wnt signaling is important in pattern specification, migration of the neural crest and is distinguished in canonical and non-canonical pathways. In the canonical pathway, binding of Wnt ligands to Frizzled receptors induces the translocation of β-catenin in the nucleus, where it engages the N-terminus of Tcf/Lef transcription factors. In absence of Wnt signal, Tcf/Lef proteins repress target genes whilst the interaction with β-catenin converts them into transcriptional activators [3-5]. Non-canonical pathways are the planar cell polarity (PCP) and calcium mobilisation *via* activation of G proteins. PCP plays an important role in directional migration of neural crest cells during development, suggesting that non-canonical Wnt signalling is a key element of this process [6]. The seven trans-membrane protein frizzled (Fzd) transduce the Wnt signal and 10 receptors have been so far identified, some of which have been shown to be involved in the development of the nervous system and stem cell signalling [7-12].

In this study, we investigated whether surface expression of frizzled receptors could be of clinical and biological relevance in neuroblastoma.

## RESULTS AND DISCUSSION

**Figure 1:**
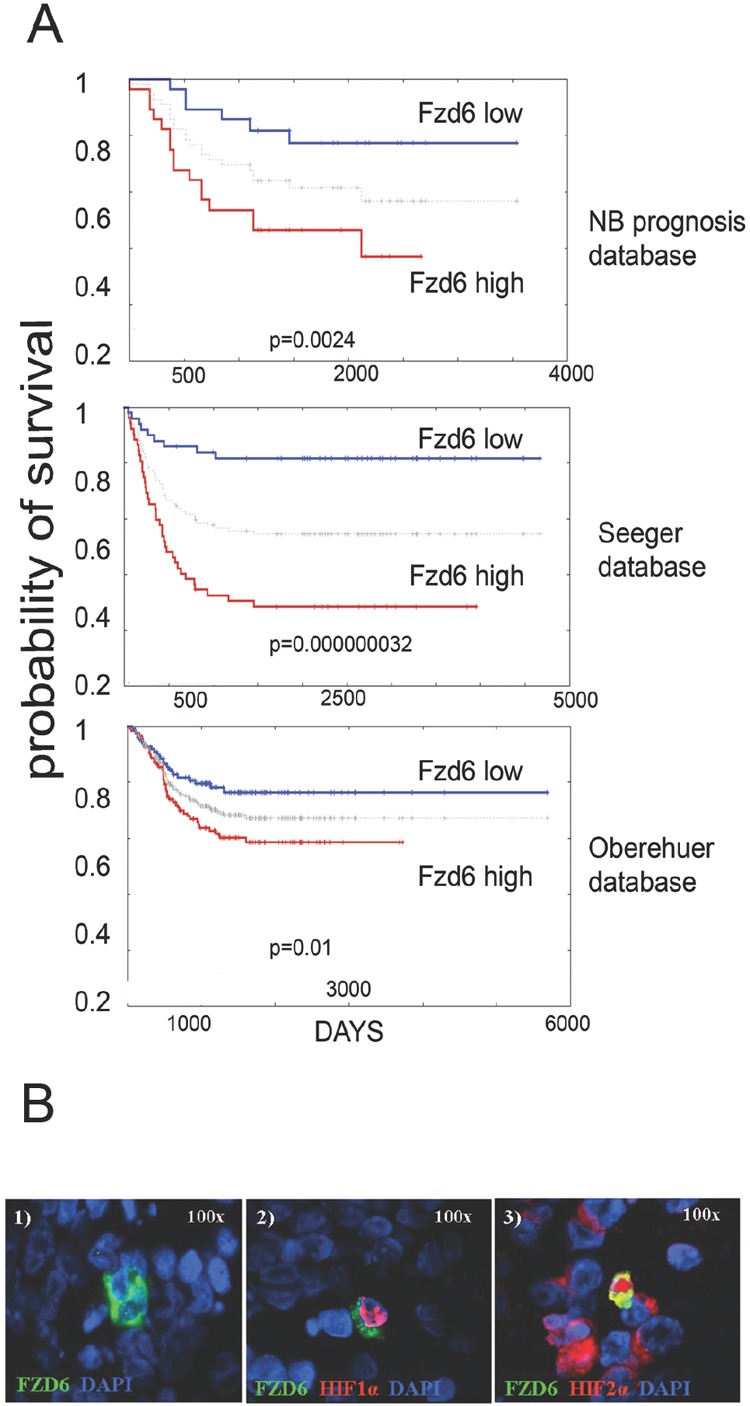
Fzd6 is associated with poor prognosis in neuroblastoma and marks cells with features of cancer stem cells A) Kaplan Meier survival estimation curves were generated using the Oncogenomics website (http://home.ccr.cancer.gov/oncology/oncogenomics). The grey line between high and low curves indicates median values. P values from log rank analysis are indicated at the bottom of each panel. B). Panel 1. Immunofluorescence analysis of Fzd6 expression in a representative metastatic primary human neuroblastoma. Green=Fzd6; Blue=DAPI. Panel 2. Immunofluorescence analysis to show co-localisation of Fzd6 and nuclear HIF1α. Green=Fzd6; red=HIF1α; blue=DAPI. Panel 3. Immunofluorescence analysis to show co-localisation of Fzd6 and nuclear HIF2α. A few Fzd6 negative cells show cytoplasmic HIF2α staining. Green=Fzd6; red=HIF2α; blue=DAPI.

**Figure 2:**
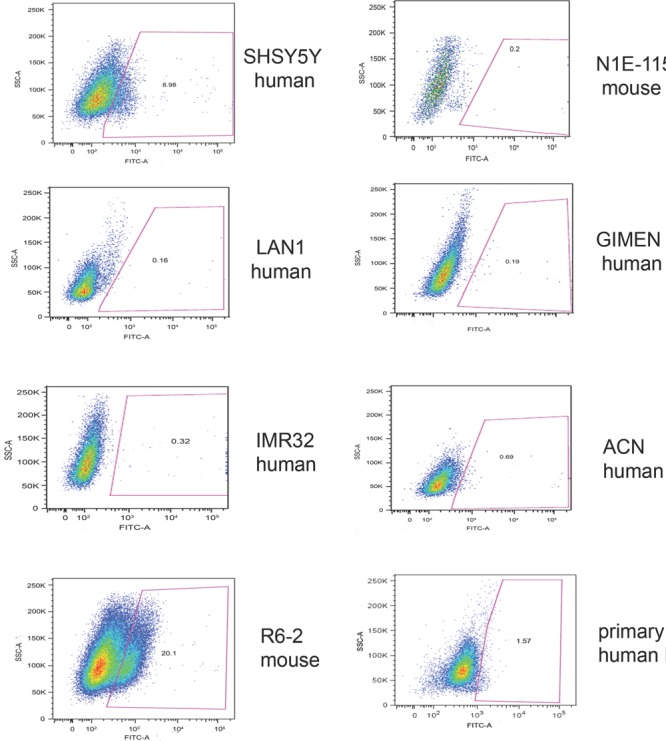
Expression of Fzd6 in primary or established neuroblastoma cell lines Cells were stained with FITC labelled Fzd6 antibodies and subjected to flow cytometry analysis to enumerate cells positive or negative to the marker. An isotype matched, irrelevant fluorescinated antibody was used as a negative control.

We initially investigated whether frizzled receptors were associated with survival of neuroblastoma patients. Using the neuroblastoma prognosis databases on the Oncogenomics repository, we found that only Fzd6, among the 10 frizzled receptors, was statistically significantly associated with poor survival in all databases (figure 1A and http://home.ccr.cancer.gov/oncology/oncogenomics). Notably, immunofluorescence analysis of tissue sections from primary human neuroblastomas showed that Fzd6 positive cells were rare (1-8 per one thousand cells, Suppl. Table 1; Fig. 1B, panel 1). All of these cells expressed HIF1α that was not detected in Fzd6 negative cells (Fig. 1B, panel 2). In addition, all Fzd6 positive cells showed prominent nuclear staining for HIF2α, indicative of transcriptionally active status. HIF2α was also detected in 60±2% Fzd6 negative cells, although with a cytoplasmic staining pattern (Fig. 1B, panel 3). These findings suggest that Fzd6 positive cells localise in tumour hypoxic areas [13]. We next assessed whether established or primary mouse and human neuroblastoma cell lines were positive to Fzd6. The latter cells were freshly disaggregated from a metastasis of a stage 4 neuroblastoma patient and short-term cultured. Only a small percentage of cells within the population of each cell line were stained with the Fzd6 antibody, further corroborating the hypothesis that Fzd6 marks a specific cell subset within the bulk of the tumour (Fig. 2).

**Figure 3:**
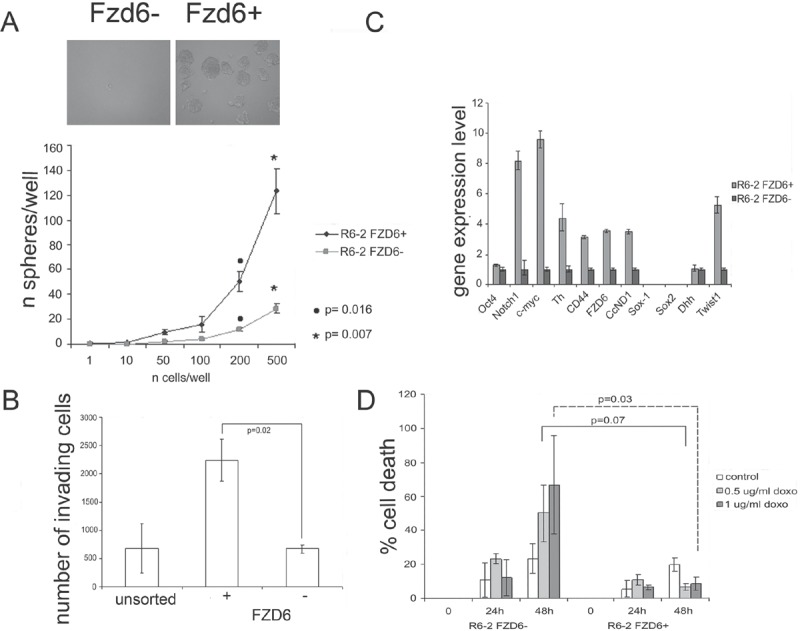
Fzd6 expressing cells display biological features of cancer stem cells A) Neurospheres assay. The number of spheres per well was plotted against the number of cells plated. Error bars indicate standard deviations, statistical significance was assessed using two-sided Student-T test (n=3). The phenotype of spheres deriving from Fzd6+ and Fzd6− cells is shown in the inset. B) *In vitro* invasion assay. Error bars indicate standard deviations, statistical significance was assessed using two-sided Student-T test (n=3). C) Gene expression analisis of Fzd6 positive and negative R6-2 neuroblastoma cells. Gene expression levels in Fzd6 negative cells were arbitrarily set to 1. CcND1 indicates cyclin D1. Error bars indicate standard deviations of triplicate wells. The experiment was repeated twice with similar results. D) Cell death assay. Mouse neuroblastoma cells were sorted as and plated in the presence or absence of the indicated concentrations of doxorubicin (doxo) for 24 or 48 hours. Cell death was scored by trypan blue dye exclusion assay. Error bars indicate standard deviations, statistical significance was assessed using two-sided Student-T test (n=3).

To investigate whether the expression of Fzd6 could be compatible with a stem cell-like phenotype, we FACS-sorted Fzd6 positive and negative cells from the neuroblastoma cell line R6-2, recently established by our group from MYCN mice [14], since these cells were enriched in Fzd6 positive cells compared to others (Fig. 2). Firstly, we observed that Fzd6-positive neuroblastoma cells formed more neurospheres in serum free medium and were more invasive than their Fzd6-negative counterparts in *in vitro* invasion assays (Fig. 3 A,B). To extend these findings, we investigated whether the positive and negative populations also differed in terms of gene expression. Fzd6 positive, compared to negative, cells presented activation of canonical and non-canonical Wnt target genes such as MYC, CD44, cyclin D1, Twist1, tyrosine hydroxylase (TH), and the neural stem cell marker Notch1. In contrast, expression of other non-Wnt target genes and stem cells markers Oct4, Sox1, Sox2 and DHH was either undetected or unchanged (Fig. 3C). A feature of neuroblastoma cancer stem cells is their ability to resist drug killing [15]. Fzd6 positive cells were significantly more resistant to doxorubicin killing than their negative counterparts (Fig. 3D). These experiments supported the hypothesis that Fzd6 positive cells, at least according to these *in vitro* assays, have a phenotype reminiscent of cancer stem cells.

**Figure 4:**
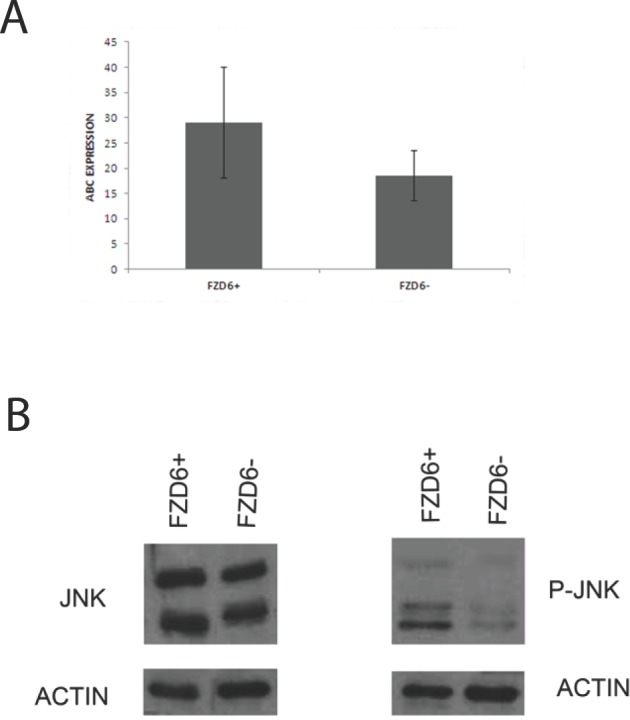
Fzd6 expression is associated to activation of the non-canonical Wnt pathway A) Fzd6 positive and negative cells were subjected to immunofluorescence staining with an antibody specific for activated beta-catenin (indicated by ABC). The average of the percentages of cells positive for the antibody in multiple fields are plotted in the y axis. The error bars indicate standard deviations. The difference is not statistically significant (p=0.1). B) western blot analysis showing the expression of total (left panel) and activated/phospho (right panel) JNK in Fzd6 positive (+) and negative (−) neuroblastoma cells.

**Figure 5:**
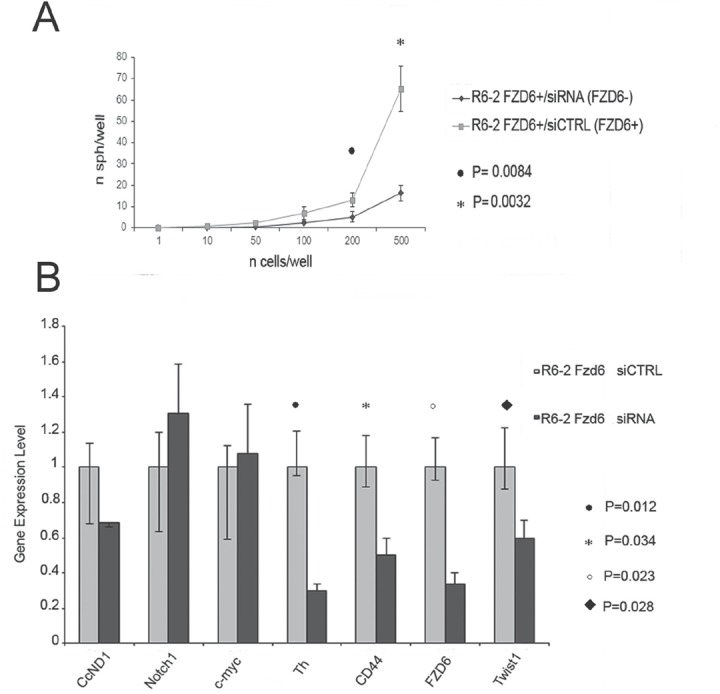
RNAi downregulation of Fzd6 inhibits neurospheres formation and expression of Wnt target genes A) Fzd6 positive R6-2 cells were separated by FACS sorting, transfected with scrambled (indicated by CTRL) or Fzd6 siRNA oligonucleotides and plated in neurospheres forming medium at the indicated concentrations. Statistical significance was assessed by two-sided T-test (n=3). B) Neurospheres obtained after transfection of R6-2 cells with scrambled or Fzd6 siRNA oligonucleotides were collected and subjected to analysis of gene expression by Real-time PCR. Gene expression levels observed in scrambled siRNA transfected cells were arbitrarily set to 1 and compared to expression levels in Fzd6 siRNA transfected counterparts. Statistically significant differences obtained in 3 independent transfections are indicated.

Frizzled receptors can activate canonical or non-canonical Wnt pathways. To distinguish between the two possibilities, we stained FACS sorted R6-2 cells with an antibody reactive against activated beta-catenin or phosphorylated JNK as a read out for activation of the canonical and non-canonical pathways, respectively. Fzd6 positive cells showed a slight enrichment of activated catenin compared to negative cells; however the difference was not statistically significant (Fig. 4A). Notably, phosphorylated JNK was strongly increased in Fzd6 positive, compared to negative, cells, indicating activation of the non-canonical pathway (Fig. 4B). In agreement with this observation, Wnt4/Fzd6 signalling was recently shown to activate the non-canonical pathway in murine haematopoietic precursor cells [16]. Fzd6 signalling is required for the stem-like phenotype of neuroblastoma cells, since neurospheres formation was drastically reduced in cells transfected with an Fzd6 siRNA (Fig. 5A). The non-canonical Wnt-target genes CD44 and TH, along with the mesenchymal transcription factor Twist1, were downregulated, whereas expression of the canonical Wnt-target genes MYC and cyclin D1 was unchanged in Fzd6 interfered cells, further corroborating the hypothesis that Fzd6 is mainly required to activate the Wnt non-canonical pathway in neuroblastoma cells (Fig. 5B). The requirement for the expression of Fzd6 for neurospheres formation was validated using an additional Fzd6 siRNA (supplementary figure 1).

**Figure 6:**
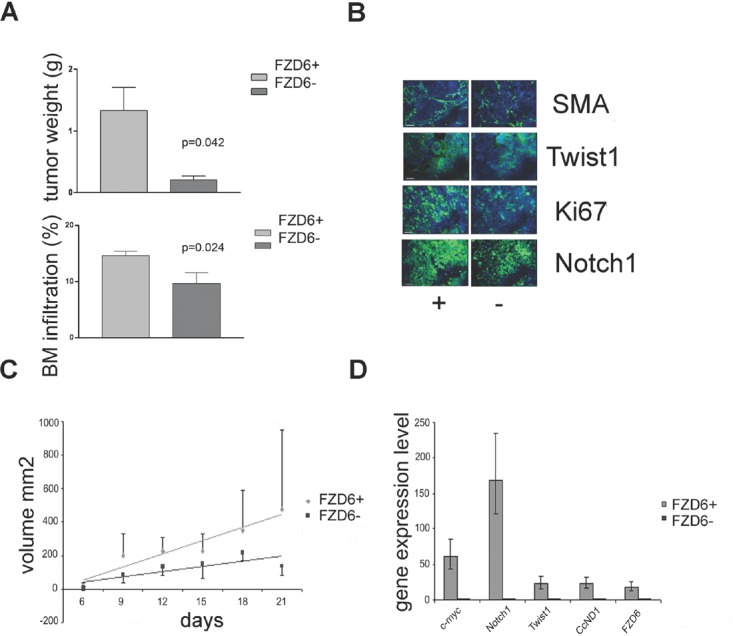
Fzd6 expression is associated with aggressive growth in vivo A) Orthotopic transplantation of Fzd6 positive or negative cells into immunodeficient mice. Upper graph, bars indicate average tumours weights and Error bars indicate standard deviations. Statistical significance was verified by a two-sided Student T test (p=0.042). Neuroblastoma cells infiltrating the bone marrow of mice was assessed by flow cytometry (FACS) after incubation with an anti-GD2 mAb (bottom graph). Error bars indicate standard deviations and the statistical significance was verified by a two-sided Student T test (p=0.024). B) Immunofluorescence analysis of tumor sections from orthotopically implanted neuroblastomas showing increased expression of the indicated markers in Fzd6 positive versus negative tumors. Bar = 100μm (SMA, KI67, Notch1) or 10μm (Twist1). Quantitative assessment is shown in supplementary figure 2. C) Xenograft transplantation of SHSY5Y cells. 1×10^6^ cells were injected in the flanks of immunodeficient SCID mice and tumor volumes were recorded at the indicated times. Regression lines represent average rates of growth. The *P* value refers to the difference in regression coefficients (Fzd6 positive R= 0.903, Fzd6 negative R= 0.658; p=0.037). D) Gene expression analysis in Fzd6 positive and negative SHSY5Y cells was conducted. Error bars indicate standard deviations of triplicate wells.

To gain insights on the role of the Fzd6 receptor in the context of a physiologically relevant human model, we used primary neuroblastoma cells freshly disaggregated from a metastasis of a stage 4 neuroblastoma patient. Fzd6 positive and negative cells were separated by FACS sorting of short-term cultures and injected orthotopically into the adrenal glands of immunodeficient mice. After 7 weeks, we observed significantly larger tumors in the adrenal glands of mice injected with Fzd6 positive cells, compared to those formed by Fzd6 negative cells. Furthermore, after 8.5 weeks there were significantly more Fzd6 positive, than negative, cells metastasising to the bone marrow of tumor injected mice (Fig. 6A). Expression of Notch1, the proliferation marker ki67 and the Wnt target gene and mesenchymal stem cell marker Twist1 were significantly increased in Fzd6 positive tumors, corroborating the hypothesis that Fzd6 marks cells with enhanced capacity to proliferate and metastasise *in vivo* (Fig. 6B; quantification of this experiment is shown in supplementary figure 2). Expression of the vascular marker α-SMA was significantly increased in Fzd6 positive, compared to negative, tumours, suggesting that Fzd6 expression is associated to a vascular phenotype (Fig. 6B and supplementary figure 2). Interestingly, one feature of neuroblastoma stem cells is their ability to directly and indirectly promote vasculogenesis [17].

In further experiments, the classical neuroblastoma cell line SHSY5Y was FACS sorted after staining with the Fzd6 antibody and injected into the flanks of immunodeficient SCID mice. Cells positive for the Fzd6 marker formed statistically significantly larger tumours than the Fzd6 negative counterparts, (Fig. 6C). Assessment of gene expression before injections into mice confirmed that Wnt-target genes and Notch1 were activated in Fzd6 positive, compared to negative, SHSY5Y cells (Fig. 6D).

A previous study showed that Fzd6 expression is up-regulated in Chronic Lymphocytic Leukemia (CLL), marking a population of leukaemic stem cells, and development of CLL is delayed significantly in Fzd6 knock out mice [12]. Moreover, Fzd6 is highly expressed in the slow-dividing fraction of human cordon blood progenitor cells, suggesting a role in self-renewal [18].

In this study, we have shown that Fzd6 expression is associated with poor prognosis of neuroblastoma patients, marking tumour cells that grow aggressively *in vivo*. Fzd6 positive neuroblastoma cells reside in hypoxic areas and express nuclear HIF2α, which was shown to mark tumour initiating cells in neuroblastomas [19]. Fzd6 positive cells display biological features that have been detected in cancer stem cells, such as the ability to form neurospheres with high efficiency, increased invasive ability and resistance to chemotherapeutic drugs. Furthermore, cells positive to Fzd6 express stem cell associated genes.

Whether Fzd6 is a genuine marker of neuroblastoma stem cells will need to be further assessed in serial transplantation experiments in which Fzd6 positive cells isolated from neuroblastoma biopsies will be injected into immunodeficient mice to recapitulate patients' tumours.

The stem cell markers CD133, CD24 and CD34 have been detected on the surface of neuroblastoma cells [20, 21] but none of these molecules is specific to the neural crest and their expression is detected in normal neural and haematopoietic stem cells. Therefore, these markers are unlikely to be used as therapeutic targets in neuroblastoma or other forms of cancer. Fzd6 expression is mainly restricted to embryonic life, suggesting that Fzd6 could be pharmacologically targeted in cancer patients to eliminate aggressive cell subsets within the bulk of tumours. Therapeutic approaches aimed at inhibiting Fzd6 positive tumor cells, if developed, could hold promise in the clinic for the treatment of aggressive forms of neuroblastoma and other tumour types showing expression of this marker.

## MATERIALS AND METHODS

### Neurospheres assay

R6-2 neuroblastoma cells from MYCN transgenic mice [14] were incubated with a Fzd6 antibody and a fluorescinated secondary antibody (both from R&D Systems, Minneapolis, MN). Cells were separated by FACS sorting and cultured in serum free medium consisting of a 3:1 mixture of DMEM:F12, (Invitrogen), containing 2% B27 supplement (Gibco), 40 ng/ml basic fibroblast growth factor 2 (bFGF) (Millipore) and 20 ng/ml epidermal growth factor (Invitrogen). Cells were seeded into microwell plates at a concentration ranging from 500 to 1 cell per well, and maintained in culture for 7 days.

### Western blot

Cells were lysate in RIPA Buffer (10 mM Tris-Cl [pH 8.0], 1 mM EDTA, 1% Triton X-100, 0.1% sodium deoxycholate, 0.1% SDS, 140 mM NaCl), protease and phosphatase inhibitors (Roche). Proteins were separated by SDS/PAGE on 10% gels, transferred to PVDF membrane (Amersham, Pharmacia), and incubated with antibodies against SAPK/JNK (Cell Signalling) or Phospho-SAPK/JNK (Thr183/Tyr185) (Cell Signaling). Immunoblots were visualised using the enhanced chemoluminescence.

### Immunofluorescence analysis of active-β-catenin

Cells were fixed in 4% paraformaldehyde and permeabilized in 0.5% TRITON-X in PBS. Blocking was carried out with 50% Normal Goat Serum (NGS) and 1% BSA in PBS. Cells were next incubated for 1 hour with 1:1000 dilution in blocking buffer of anti-active-β-catenin (anti-ABC, clone 8E7, Millipore). After incubation with a rhodamine labeled secondary antibody, the slides were counterstained with Dapi (Roche).

### In vitro invasion assay

Mouse neuroblastoma cells positive or negative for Fzd6 were separated by FACS sorting and plated on the top well of invasion chambers in triplicate. The number of cells invading the bottom chamber was scored after staining with crystal violet.

### Quantitative PCR

Quantitative real-time PCR was performed using Taq-Man master mix (Applied Byosistems) and a 7900HT Fast Real-Time PCR (Applied Biosystems). cDNAs were amplified using murine TaqMan Gene Expression Assays for the following genes: Oct-4 (Mm03053917_g1), Notch1 (Mm01326752_g1), c-myc (Mm00487804_m1), Th (Mm00447549_g1), CD44 (Mm01277160_m1), Frizzled 6 (Mm00433383_m1), CcD1 (Mm03053889_s1), Sox1 (Mm01209749_s1), Sox2 (Mm03053810_s1), Dhh (Mm03053542_s1), Twist (Mm00442036_m1), HPRT1 (Mm01545399_m1). The human TaqMan gene expression assays were: Myc (Hs00905030_m1), Notch1 (Hs0162014_m1), Twist1 (Hs00361186_m1), CCND1 (Hs00765553_m1), Fzd6 (Hs00171574_m1), GAPDH (Hs99999905_m1).

## Supplementary Materials

Supplementary Figures

Supplementary Table

Supplementary Methods
