# Supplementary material for: Frizzled receptor 6 marks rare, highly tumourigenic stem-like cells in mouse and human neuroblastomas
Source: Oncotarget. 2011 Dec 31;2(12):976–83. doi: 10.18632/oncotarget.410 (PMC3282103; doi:10.18632/oncotarget.410)
Supplement: Supplementary file 1 — Supplementary Figures [file oncotarget-02-976-s001.pdf]

Supplementary Table 1: Clinical and biological characteristics of the neuroblastoma patients analysed in the study.

**Pz**	**Stage**	**Age**	***MYCN***	**Relapse**	**Outcome**	**Fzd 6$^+$ cells**
NB1	1	Infant	N	Y	CR	4/1000
NB2	1	Infant	N	Y	CR	2/1000
NB3	2A	Infant	Y	Y	DOD	1/1000
NB4	2A	Infant	N	N	CR	1/1000
NB5	2B	Non-infant	N	Y	CR	2/1000
NB6	2B	Non-infant	N	Y	DOD	2/1000
NB7	3	Infant	N	N	CR	2/1000
NB8	3	Non-infant	Y	N	AWED	4/1000
NB9	3	Infant	Y	N	CR	2/1000
NB10	3	Non-infant	Y	N	CR	8/1000
NB11	3	Non-infant	Y	Y	DOD	1/1000
NB12	4	Non-infant	N	N	CR	2/1000
NB13	4	Non-infant	Y	Y	DOD	3/1000
NB14	4	Non-infant	Y	N	DOD	1/1000
NB15	4	Infant	Y	Y	DOD	2/1000
NB16	4	Non-infant	Y	Y	DOD	2/1000
NB17	4	Infant	Y	N	CR	2/1000
NB18	4	Non-infant	Y	Y	DOD	2/1000

Abbreviations: N, no; Y, yes; AWED, alive with evidence of disease; DOD, dead of disease; CR, complete remission.
