# Supplementary material for: Frizzled receptor 6 marks rare, highly tumourigenic stem-like cells in mouse and human neuroblastomas
Source: Oncotarget. 2011 Dec 31;2(12):976–83. doi: 10.18632/oncotarget.410 (PMC3282103; doi:10.18632/oncotarget.410)
Supplement: Supplementary file 2 — Supplementary Table [file oncotarget-02-976-s002.pdf]

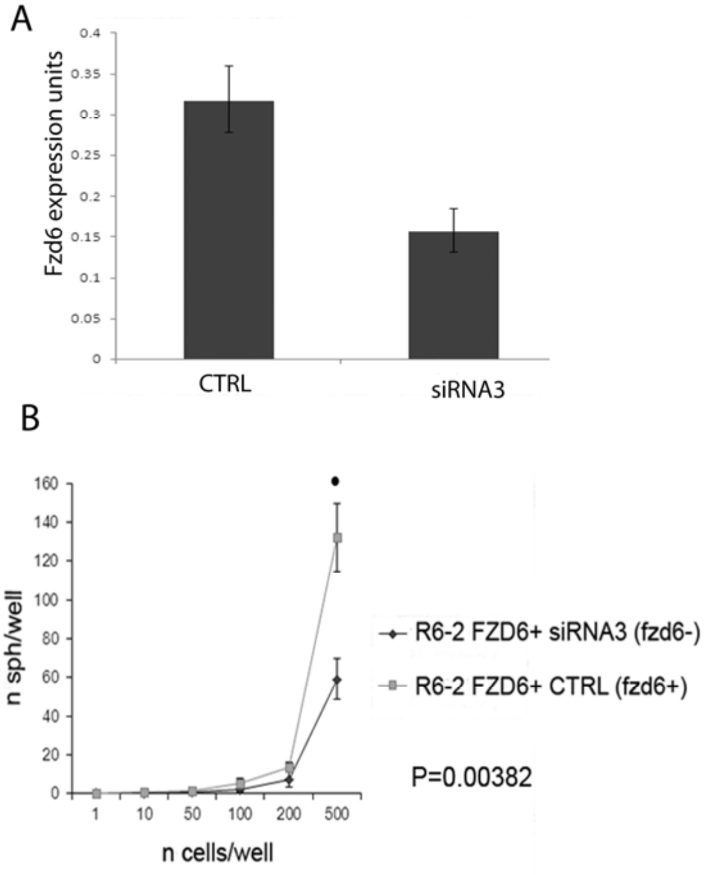

Supplementary Figure 1: Inhibition of Fzd6 expression with a further siRNA validates its role in neurospheres formation.

A) Expression of Fzd6 mRNA was assessed by Real-time PCR after transfection of a scrambled control (CTRL) or Fzd6-specific (siRNA3) siRNA into R6-2 cells.

B) Neurospheres assay. The number of spheres per well was plotted against the number of cells plated. Error bars indicate standard deviations, statistical significance was assessed using two-sided Student-T test (n=3).

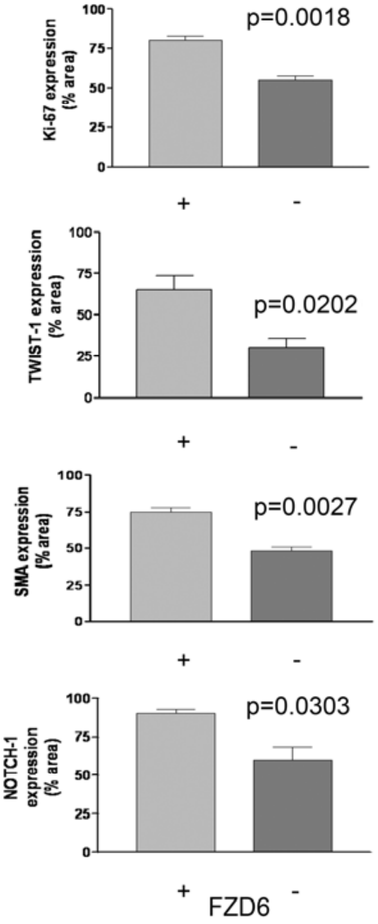

Supplementary Figure 2: Quantification of the immunofluorescence analysis of markers expression in orthotopically transplanted tumors.

Paraffin-embedded tissue sections were de-paraffinised by a xylene-ethanol sequence, re-hydrated in a graded ethanol solutions, and TRIS-buffered saline (TBS, pH 7.6), and then processed for antigen retrieval by boiling tissue sections for 10 min in 1 mM EDTA, pH 8.0, in a microwave oven. The sections were then washed twice in PBS and saturated with 2% BSA in PBS before staining with primary antibodies against: KI67 proliferation antigen (mouse anti-human KI67,

clone MIB-1, Dako); smooth muscle actin (SMA, mouse anti-human, clone 1A4, Dako); TWIST1 (rabbit anti-Twist1, Sigma-Aldrich); Notch1 (monoclonal anti-Notch1, clone 3E12, Sigma-Aldrich). Binding of the primary antibodies was detected with green FITC-conjugated goat either anti-mouse or anti-rabbit (Caltag Laboratories-Invitrogen) immunoglobulin for immunofluorescence analyses. Specimens were analyzed on a Nikon E-1000 fluorescence microscope equipped with specific filters for FITC and 4',6-diamidino-2-phenylindole (DAPI). Morphometric analysis was performed on 9 randomly selected fields every 3 sections, using Image Analysis software (Olympus Italia).
