# Supplementary material for: Frizzled receptor 6 marks rare, highly tumourigenic stem-like cells in mouse and human neuroblastomas
Source: Oncotarget. 2011 Dec 31;2(12):976–83. doi: 10.18632/oncotarget.410 (PMC3282103; doi:10.18632/oncotarget.410)
Supplement: Supplementary file 3 — Supplementary Methods [file oncotarget-02-976-s003.pdf]

Supplementary Materials and Methods

Immunofluorescence analysis in neuroblastoma patients

Eighteen neuroblastoma (NB) tissue sections were collected from patients untreated at diagnosis. Two patients had stage 1 disease, four stage 2, five stage 3 and seven stage 4 according to the International Neuroblastoma Staging System classification. Each tumor sample contained at least 80% malignant cells as assessed by histological analyses. Studies with human tissues were approved by a local ethical committee. Formalin-fixed, paraffin-embedded tissue sections (2 to 4µm) were blocked with PBS + 2% bovine serum albumin for 30 mim. Next, slides were incubated with anti-Frizzled 6 antibody (diluted 1/50 Novus Biologicals, Littleton, CO,USA) , Anti HIF1α (1/100, Novus biologicals) anti HIF2α (1/100, Novus Biologicals) overnight at 4°C. AlexaFluor 488-conjugated goat anti-rabbit and/or AlexaFluor 568-conjugated goat anti mouse antibodies (diluted 1/200; Invitrogen Corp., CA, USA) were added as secondary reagents for 1 hour at room temperature. After washings in PBS, slides were counterstained with antifade solution with 4'-6-diamidino-2- phenylindole (DAPI) (Vector, Burlingame, CA). The slides were then analyzed using a Nikon E-1000 fluorescence microscope (Nikon Instruments, Tokyo, Japan) equipped with appropriate filter sets and Genikon imaging system software (Nikon Instruments).

Orthotopic injections

A surgically resected human neuroblastoma metastasis was obtained with the written consent of the child's family. Cells were washed in PBS, mechanically dispersed and passed through 70µm cell strainer (BD Biosciences) to obtain a single-cell suspension. Cells were plated in 25ml tissue culture flasks and cultured in RPMI 1640 medium (supplemented with 20% FCS, 2 mM l-glutamine, 0.1 mM 2-mercaptoethanol, 1 mM sodium pyruvate, 1× nonessential amino acids and 1% penicillin/streptomycin solution). FACS analysis with a GD2 antibody showed that >90% of cells were positive to this neuroblastoma antigen. Five/six-week-old NOD-SCID mice (12 mice/group) were anaesthetised with ketamine, subjected to laparotomy, and injected with FACS sorted Fzd6 positive or negative cells (5×10^5 cells in 10 µL saline solution) in the capsule of the left adrenal gland. Mice (n=3) were killed after 7 weeks and the weight of tumours was recorded with the aid of a precision balance. The remaining mice (n=10) were sacrificed after 8.5 weeks and neuroblastoma cells in the bone marrows were enumerated by staining with a GD2 antibody and flow cytometry.
